# Network analysis of pig movement data as an epidemiological tool: an Austrian case study

**DOI:** 10.1038/s41598-023-36596-1

**Published:** 2023-06-14

**Authors:** Gavrila A. Puspitarani, Reinhard Fuchs, Klemens Fuchs, Andrea Ladinig, Amélie Desvars-Larrive

**Affiliations:** 1 Unit of Veterinary Public Health and Epidemiology, University of Veterinary Medicine Vienna, Veterinaerplatz 1, 1210 Vienna, Austria; 2 Complexity Science Hub Vienna, Josefstaedter Strasse 39, 1080 Vienna, Austria; 3 Department for Data, Statistics and Risk Assessment, Austrian Agency for Health and Food Safety (AGES), Zinzendorfgasse 27/1, 8010 Graz, Austria; 4 Institute of Systems Sciences, Innovation and Sustainability Research, University of Graz, Merangasse 18/1, 8010 Graz, Austria; 5 University Clinic for Swine, University of Veterinary Medicine Vienna, Veterinaerplatz 1, 1210 Vienna, Austria; 6 VetFarm, University of Veterinary Medicine Vienna, Kremesberg 13, 2563 Pottenstein, Austria

**Keywords:** Epidemiology, Infectious diseases, Computational science

## Abstract

Animal movements represent a major risk for the spread of infectious diseases in the domestic swine population. In this study, we adopted methods from social network analysis to explore pig trades in Austria. We used a dataset of daily records of swine movements covering the period 2015–2021. We analyzed the topology of the network and its structural changes over time, including seasonal and long-term variations in the pig production activities. Finally, we studied the temporal dynamics of the network community structure. Our findings show that the Austrian pig production was dominated by small-sized farms while spatial farm density was heterogeneous. The network exhibited a scale-free topology but was very sparse, suggesting a moderate impact of infectious disease outbreaks. However, two regions (Upper Austria and Styria) may present a higher structural vulnerability. The network also showed very high assortativity between holdings from the same federal state. Dynamic community detection revealed a stable behavior of the clusters. Yet trade communities did not correspond to sub-national administrative divisions and may be an alternative zoning approach to managing infectious diseases. Knowledge about the topology, contact patterns, and temporal dynamics of the pig trade network can support optimized risk-based disease control and surveillance strategies.

## Introduction

Social networks provide a conceptual framework for representing relations between elements of a complex system^1,2^. Social network analysis (SNA) is the process of investigating social structures through the use of graph theory, which provides vocabulary and mathematical operations to study the properties of social networks^1,3,4^. Livestock movement patterns (e.g. where, when, and how many farm animals move between premises and markets) can be studied through network-based approaches. A livestock trade network consists of nodes or vertices (e.g. farms, markets, or slaughterhouses) and edges that connect pairs of nodes and represent the nature (i.e. movement of animals from a holding to another one) and direction of the relationship between the connected nodes. Moreover, edges can have weights, i.e. values that characterize the connection between a pair of nodes, e.g. number of animals or frequency of exchanges^3,5,6^.

Leveraging lessons learned from the epidemics of bovine spongiform encephalopathy (BSE) in Europe in the 1990s^7–9^ and foot-and-mouth disease (FMD) in the UK in 2001^10–12^, several EU countries have developed livestock registration and movement databases that facilitate tracing of animals and response to outbreaks^13,14^. More recently, the Animal Health Law (AHL, Regulation (EU) 2016/429) sets out requirements for its member states regarding the electronic identification and registration of animals and certain animal products as well as rules for notifying and recording their movements within the EU^15^. Broader availability and enhanced quality of livestock movement data in the EU, together with more accessible computing power and analytical software, have encouraged the study of real-world complex networks in veterinary epidemiology, enabling new insights into the complexity of livestock trade and dynamics of infectious animal disease transmission^16,17^. The analysis of livestock movement data based on graph theory, e.g. in the UK^12,18,19^, Denmark^20,21^, Germany^22^, Spain^23^, France^24,25^, Italy^26^, Brazil^27^, and Ireland^28^ proved to be a valuable tool to explore the dynamics of trade patterns between livestock operations, quantitatively characterize the topology of animal trade networks, and understand the role each animal holding plays in the network^3,29^. In epidemiology, network approaches revealed also very powerful, e.g. to model patterns of infectious disease spread, risk of infectious disease introduction, estimate outbreak sizes^3,12,18,21–23,30–35^, develop and evaluate disease surveillance and mitigation strategies^29,36,37^, or facilitate policy development^6,31,38^.

However, many studies propose a static representation of the livestock trade network, neglecting the temporal component of it^18^, which oversimplifies the reality and presents major limitations for the understanding of disease dynamics^18,22,32,33,39–41^. Similarly, when applied to animal trade networks, community detection, also known as graph partitioning or clustering, have generally not exploited the temporal features of the network^12,38,41–45^. The purpose of community detection algorithms is to unveil subgroups of highly connected nodes within the network that share specific properties^46^. Community detection is a relatively recent field of research^47^ and there is a lack of agreement on the optimal detection method^48,49^, which may explain why it is often ignored in the analysis of animal trade networks. However, taking into account the temporal dynamics of the community structure in livestock networks could reveal temporal variations in the strength of the connections between a group of holdings as well as changes in the trade preferences over time (e.g. number and size of communities)^48^.

In 2021, Austria exhibited 103% of self-sufficiency in pork meat production^50^. The same year, the pork consumption reached 34.2 kg per capita, making pork the most consumed meat in the country^51,52^. However, the Austrian pig value chain is threatened by major exotic infectious diseases such as African swine fever (ASF), classical swine fever (CSF), and FMD, which can be introduced, for example, through live animal trade or importation of pork products^53^. Furthermore, endemic infectious pathogens, e.g. the porcine reproductive and respiratory syndrome (PRRS) virus^54^, *Mycoplasma hyopneumoniae*^55^, porcine circovirus type 2 (PCV2)^56^, *Streptococcus suis*^57^, and *Actinobacillus pleuropneumoniae*^58^ often lead to a reduction in the performance of infected animals and induce an important, yet often underestimated, burden on production as well as on animal health and welfare^59^. Despite these risks and potential impact on the pig sector, to date, data on pig movements in Austria has not been analyzed for scientific purposes. It is essential to understand the intrinsic structure of the Austrian pig movement network and translate mathematical results into information that could support evidence-informed surveillance systems for early detection of outbreaks as well as efficient, timely response to infectious disease outbreaks^3,30,60^.

In this study, we aimed to evaluate the structural characteristics and dynamic patterns of the Austrian pig movement network and discuss their potential applications in veterinary epidemiology. More specifically, we intended to:

- Describe the Austrian pig trade network topology and trends over a seven-year period;
- Apply a community detection algorithm to uncover hidden relationships among nodes (holdings) in the network and investigate community temporal dynamics;
- Generate meaningful information that could be concretely used, e.g. in risk analysis or disease surveillance and control planning.

## Methods

### Data

In Austria, data on animals, livestock movements, and holdings (e.g. farms, markets and slaughterhouses) are recorded in the Verbrauchergesundheits Information System (VIS) or *Consumer Health Information System*, an electronic database of the Austrian Federal Ministry of Social Affairs, Health, Care and Consumer Protection (BMSGPK) and operated by Statistics Austria^61^. Data used in this study covers the period January 2015 to December 2021 and consists of (1) data on registered pig-related holdings and (2) daily records of pig movements within Austria as well as to and from other countries (intra-EU trade, export and import).

For each animal holding, the data includes a unique anonymized identification number (ID), the randomized 5 km-radius geo-coordinates, information on the type of activities of the holding, and the number of pigs per production stage for each year the holding was operating, as reported yearly on a mandatory basis by the animal owner. There are four major pig production stages, themselves subdivided on the basis of the animal sex and weight: (1) breeding animals weighting > 50 kilograms (kg): gilts, sows, and boars; (2) piglets: up to 8 kg, 8–20 kg, and 20–32 kg; (3) young pigs weighting 32–50 kg; and (4) fattening pigs: 50–80 kg, 80–110 kg, and > 110 kg.

Regarding pig holding activities, the following labels (and sub-labels) are registered in the database: (1) farm (including breeding, piglet rearing, and fattening farms); (2) private pet owner; (3) processing plant (including meat and by-product processing), (4) boar station; (5) trade and logistics (including trading stable, transporter, and animal dealer), (6) collection point or animal gathering; and (7) slaughterhouse. One holding (identified by an ID) can be registered with one or more labels.

Each record of pig movement contains information on the source (ID of the holding of origin) and target (ID of the receiving holding), date of the event, number of pigs moved (hereafter called “batch”), and type of movement (see below).

### Data cleaning and pre-processing

Before using the VIS data for the analyzes, it underwent a cleaning process. For each pig batch movement, a double notification system is in place. This means that each movement is reported twice, from the holding of origin and from the receiving one. Therefore, each duplicate has been reduced to a single data row.

Each reporting holding indicates whether the movement of pigs is outgoing, ingoing, or intended for slaughter and, in the case of a foreign movement, the ISO code of the country is provided. This results in four types of movement, (1) *domestic*, i.e. movement of live pigs between holdings within Austria, (2) *slaughter*, i.e. movement of pigs that are intended for slaughter, (3) *abroad*, i.e. import or export of live pigs, and (4) *abroad-slaughter*, i.e. import of live pigs that are intended for slaughter.

Our final data included 40,951 pig holdings and 1,917,584 movement records over the period 2015–2021. The data showed animal trades between Austria and 18 EU/EEA and seven non-EU/EEA countries.

Since the study focused on revealing the structural properties of the Austrian pig trade network, movements to and from abroad were removed from the dataset. Similarly, establishments such as boar stations and processing plants were ignored. Ultimately, only “active” holdings, i.e. counting at least one trade event over the considered study period, were included in the analysis.

Our analysis assumed that the data accurately reflected the actual pig movements and that each of them corresponded to a direct transport of pigs, e.g. farm-to-farm or farm-to-slaughterhouse. Therefore, because measures applied to slaughterhouses have a very limited impact on infectious disease spread in the network^22,33,62,63^, slaughter-type movements were not included in the main analysis. After removing abroad- (0.48% of all edges) and slaughter- (59%) type of movements, therefore keeping domestic movements only, the final network included 38,888 nodes (95% of all nodes) and 783,460 edges (40.8%). In the network, each node represents a holding, regardless of its activity or label.

We plotted the spatial distribution of the holdings in QGIS software^64^ using the geo-coordinates data. We then extracted for each holding the administrative divisions, i.e. federal state and district, using shapefiles of the Austrian administrative boundaries^65^ (there are nine federal states and 94 districts in Austria). Next, we displayed the yearly farm density by counting the number of farm-labeled holdings with recorded movements within 5-km hexagonal grids for each year.

### Network representation and yearly-aggregated networks

The pig trade network was represented as a temporal directed graph, $G=(V,E)$, where *V* represents a set of nodes (pig holdings) and *E* represents a set of directed edges (movements). Each edge is characterized by $(i\xrightarrow \,j,t)$ and connects a specific node *i* (source) to a specific node *j* (target) at time *t*. The presence of $i\xrightarrow \,j$ does not necessarily imply that $j\xrightarrow \,i$ exists.

We used a static representation of the directed network, where edges were aggregated over seven years, to study the intra- and inter-state trade frequency.

To study major changes in the structure of the pig trade network *G* over the period 2015–2021, we computed a time-ordered sequence of yearly snapshots, $G=G_1,\ldots ,G_T$, with $1 \le T \le 7$, by aggregating the daily networks over one-year intervals (based on the reported date of each event). The time-ordered sequence can be considered as a multi-layer network in which the layers have a specific order in time^1^. Each layer is a directed multigraph, where more than one edge may exist between a pair of nodes^3^ (when several animal movements on different days occurred within one year). Each multigraph was converted into a digraph by collapsing the multiple edges into single-weighted ones, with edge weight corresponding to the trading frequency between pairs of nodes. We characterized the yearly networks using the metrics listed in Table 1 along with their formal definition and epidemiological relevance. Node-level measures (i.e. degree and betweenness) were calculated at graph-level through a centralization method^3^, from the centrality scores of the nodes. For each snapshot, we described its degree distribution, $p_k$, defined as the set of probabilities, *P*(*k*), that a node chosen at random will have degree *k*^1^. Additionally, for each snapshot, we searched for the presence of cycles, i.e. paths that start and end at the same node^3^.

On average, there were 24,393 $\pm \ 2354$ (SD) nodes with trading activity and 111,923 $\pm \ 8930$ edges per year (Table 2), showing a median and a mode of 16 (min. = 1; max. = 3751) and two pigs per batch, respectively. Each year, on average, 98.3% of the nodes reporting trade activity had farm label (min. = 97.8%; max. = 99.2%), 6.5% had slaughterhouse label (6.3–6.8%), 1.3% were labelled as trade and logistics facilities (1.3–1.4%), 0.11% as private pet owners (0.01–0.20%), and 0.18% as collection points (0.16–0.21%). A node can have more than one label (the proportion of nodes with more than one label in the seven-year aggregated network was 5.9%). Holdings with at least one label “farm” will thereafter be referred to as “farms”.

### Trend and seasonal patterns in the pig trade network

We computed a time series of monthly directed networks by aggregating the daily networks over each month, in which edge weight corresponded to trading frequency between pairs of animal holdings. The method of classical time series decomposition with two-sided moving averages (MA)^66^ was used to analyze the trend in the number of nodes that recorded movements, edges, and volume of traded pigs.

We additionally explored the weekly patterns (Monday to Sunday) of the pig trade network using a daily aggregation of the number of nodes, edges, and volume of traded pigs. We then performed ANOVA (analysis of variance), taking into account the interaction $weekday*month$, to test whether the average number of nodes, edges, and volume of traded pigs for each weekday differed among months. For instance, we evaluated whether the average number pigs traded on Monday over the seven-year study showed significant difference between months. Prior to the analysis, the assumptions for ANOVA were tested, e.g. we checked the normality assumption by analyzing the model residuals through a QQ plot and we checked the homogeneity of the variance assumption using a residual versus fits plot. The observations were considered independent.

**Table 1:** Theoretical definitions of the network measures and metrics used in this study and relevance for livestock movements and veterinary epidemiology.

Metrics	Theoretical definition	Epidemiological relevance
Assortativity (mixing pattern)	The assortativity coefficient measures the proportion of links between and within groups of nodes sharing identical attributes. Its value lies between $-1$ and +1, negative values mean that nodes are likely to connect with other nodes showing different attributes; a positive value shows homophily (i.e. assortative mixing)^98^.	Reflects the preferential (trade) connections of an epidemiological unit, e.g. a holding or a district, with other epidemiological units which have a similar attribute, e.g. number of trade links (degree assortativity) or location (district assortativity and federal state assortativity)^99,100^.
Average path length	The average number of steps along the shortest paths for all possible pairs of nodes. The shortest path is the smallest number of edges from node *i* to node *j*^1^.	The average number of steps needed for an infection to spread from a random holding to another randomly chosen holding^6^.
Betweenness centrality	For a node *k*, the number of shortest paths between nodes *i* and *j* that pass through *k*^1^. Captures the capacity of a node to propagate an information or infection across the network^31^.	Holdings with high betweenness can act as gatekeepers for controlling or altering the spread of pathogens; removing these holdings will fragment the network^6^.
Degree centrality	For a node, the number of edge(s) connected to it, i.e. the number of neighbor(s) it has. In directed graphs, each node has both, an in-degree, $k^{in}$ (number of incoming edges) and an out-degree, $k^{out}$ (number of outgoing edges). Nodes with a high degree, compared to other nodes in the network, are considered well connected and called *hubs*^1,6,101^.	For a holding, the numbers of sources (or trading partners) providing animals (in-degree) or that receive animals from it (out-degree)^6^. Livestock holdings with a high degree centrality are at higher risk of infection and more likely to infect a large number of other holdings in the network^81^.
Density (connectance)	Proportion of edges, among the maximum possible number of edges in the network, that are actually existing. The proportion ranges from 0 to 1, where 0 means that all nodes are isolated while a network with density of 1 displays maximal cohesion^3^.	Represents the fraction of all possible trades among all livestock holdings that are actually present^6^.
Diameter	Length of the longest shortest path, i.e. among all shortest paths between every pair of nodes in the network for which a path exists, the diameter is the length of the longest one. The smaller the diameter, the more connected the network is^1^.	Longest geodesic distance between any pair of holdings in the network. A shorter diameter means that the number of generations for a disease to spread throughout the network is reduced^6,100^.
Global clustering coefficient (CC)	Fraction of closed triplets, i.e. three nodes linked to each other and forming a closed triangle, among all possible triplets. It lies between 0 and 1^3,5^.	Reflects holding-to-holding interactions (or trade links). High clustering coefficient induces a fast spread of diseases in the network^5^.
Range	For a node, the number of nodes that can be reached from it through a path of random length^22^.	Measure the potential of a holding to spread an infectious disease in the network^22^.
Strongly connected component (SCC)	A strongly connected component is the subset of nodes in a directed graph in which a directed path exists in both directions between every pair of nodes in the subset^1^.	A subset of holdings in a livestock network in which all holdings are mutually accessible by following the direction of the trades^6^. The size of the largest SCC can be used to estimate the lower bound of the maximum epidemic size^12^.
Weakly connected component (WCC)	In a directed graph, a subset of nodes in which every pair of nodes is connected by one or more paths, ignoring the direction of edges^1^.	A subset of holdings in which a link exists between every pair of holdings, ignoring trade direction. The size of the WCC can be used to estimate the upper bound of the maximum epidemic size^12^.

**Table 2:** Summary of the graph-level metrics for the yearly snapshots of the Austrian pig trade network, 2015–2021.

	2015	2016	2017	2018	2019	2020	2021
Number of edges	126,092	119,554	115,602	109,350	105,618	106,917	100,327
Number of nodes	27,938	26,752	25,348	23,860	22,609	22,578	21,667
Number of animals	4,614,349	4,483,219	4,510,794	4,413,947	4,404,170	4,493,835	4,373,382
Number of farms	27,498	26,268	24,909	23,667	22,109	22,163	21,263
Assortativity (degree)	−0.09	−0.09	$-$0.08	$-$0.09	−0.09	−0.10	−0.09
Assortativity (state)	0.87	0.87	0.86	0.87	0.85	0.84	0.84
Assortativity (district)	0.45	0.45	0.44	0.44	0.43	0.43	0.42
Average shortest path length	10.1	8.8	10.7	7.1	8.0	6.2	6.2
Betweenness centrality	0.01	0.01	0.03	0.01	0.01	0.00	0.01
Diameter	29	27	28	23	22	19	25
Degree (total)	0.01	0.01	0.02	0.02	0.02	0.02	0.02
Degree (in-)	0.01	0.01	0.01	0.03	0.01	0.01	0.01
Degree (out-)	0.03	0.03	0.03	0.03	0.03	0.03	0.03
Edge density	$5.7 \times 10^{-5}$	$5.8 \times 10^{-5}$	$6.2 \times 10^{-5}$	$6.5 \times 10^{-5}$	$6.8 \times 10^{-5}$	$6.9 \times 10^{-5}$	$7.1 \times 10^{-5}$
Global CC	$4.4 \times 10^{-3}$	$3.9 \times 10^{-3}$	$4.3 \times 10^{-3}$	$4.3 \times 10^{-3}$	$4.7 \times 10^{-3}$	$4.5 \times 10^{-3}$	$5.4 \times 10^{-3}$
Size LSCC (No. nodes)	558	646	964	210	498	127	168
Size LSCC (% of nodes)	2.0%	2.4%	3.8%	0.9%	2.2%	0.6%	0.8%
Size LWCC (No. nodes)	27,144	25,957	24,504	22,966	21,807	21,933	20,904
Size LWCC (% of nodes)	97.2%	97.0%	96.7%	96.2%	96.4%	97.1%	96.5%

CC: clustering coefficient; LSCC: largest strongly connected component; LWCC: largest weakly connected component. Farm: farm-labeled holdings.

**Table 3:** Average community matching values (SD: standard deviation) computed to compare communities detected in 2015 (as reference) with communities in each subsequent year in the Austrian pig trade network and year-over-year average community matching values, 2015–2021.

Comparison	Average community matching value ± SD
2015 (as reference)	
2015–2016	0.80 ± 0.36
2015–2017	0.70 ± 0.4
2015–2018	0.57 ± 0.41
2015–2019	0.65 ± 0.34
2015–2020	0.69 ± 0.37
2015–2021	0.71 ± 0.33
Year-over-year	
2015–2016	0.80 ± 0.36
2016–2017	0.79 ± 0.35
2017–2018	0.64 ± 0.37
2018–2019	0.67 ± 0.34
2019–2020	0.74 ± 0.33
2020–2021	0.83 ± 0.27

### Dynamic community detection

Using the yearly snapshots of the network, $G=G_1,\ldots ,G_T$, we spatially aggregated all nodes located in the same district. Edges were aggregated accordingly, so that each (directed) edge represented the frequency of trade between two districts. The node of origin and destination of each movement was therefore identified with the district name. Loops (i.e. trades that start and end in the same district) were removed. To detect groups of districts sharing similarities in connectivity patterns, so called a community, we applied the InfoMap algorithm^67^ to each yearly snapshot sequentially, i.e. from the earliest to the latest one. The algorithm decomposes the network into modules by mapping the probability flows induced by the network structure through a large number of random “surfing” processes that consider the weight and direction of the network edges. A random “surfer” differs from a random “walker” by the introduction of a teleportation probability in the random walk, which represents a positive probability that the process jumps randomly to any other node in the network. The algorithm does not allow overlap between community, i.e. a district can belong to one community only^67^.

To evidence temporal changes in the communities, we compared how similar the communities mapped at time *T* were with those at *T*+n^68^. More specifically, we defined *C* and $C'$ as a community at time *T* and *T*+n, respectively, and computed a matching value (*C*, $C'$) based on the nodes’ membership, as described in Hopcroft et al.^68^. The matching value ranges from 0 to 1; a matching value of 1 signifies that a community is identical at time *T* and *T*+n, whereas if it is equals 0, both communities display completely different members. We evaluated every community at time *T* against its corresponding community at time *T*+n. We considered $n=1$ to compare each community between consecutive years (year-over-year comparison) and $1\le n \le T-1$ to compare each community detected in the first snapshot (2015), used as a reference, with the subsequent ones (2016–2021), i.e. 2015 versus 2016, 2015 versus 2017, etc. Moreover, by averaging the matching values over all communities, we calculated a global matching value in every time window to observe the overall stability of the communities in the Austrian pig trade network over the study period.

### Additional analyses

We acknowledge that there might be movements or events that were not captured by the data, e.g. movements involving truck sharing, where pigs from different farms are collected and delivered to one or several farms or to the slaughterhouse, which represents a risk of disease spread via fomites^69^. Therefore, to provide a full picture of the Austrian pig trade network, we used the above-mentioned methods and analyzed the data including both domestic and slaughter-type movements. Outputs of these analyses (metrics of the yearly-aggregated networks and dynamic community detection) are presented in Supplementary Results.

### Software and packages

Analyses for this study were performed with R programing software^70^ (v.4.1.3) using RStudio^71^ (v.2022.07.1). The network analysis was conducted using the ’igraph’ package and the package ’TSstudio’ was used for trend analysis. Map visualization was performed in QGIS^64^ (v.3.26.3-Buenos Aires) and network visualization was executed in Gephi^72^ (v.0.9.7).

## Results

### Data description

**Figure 1:**
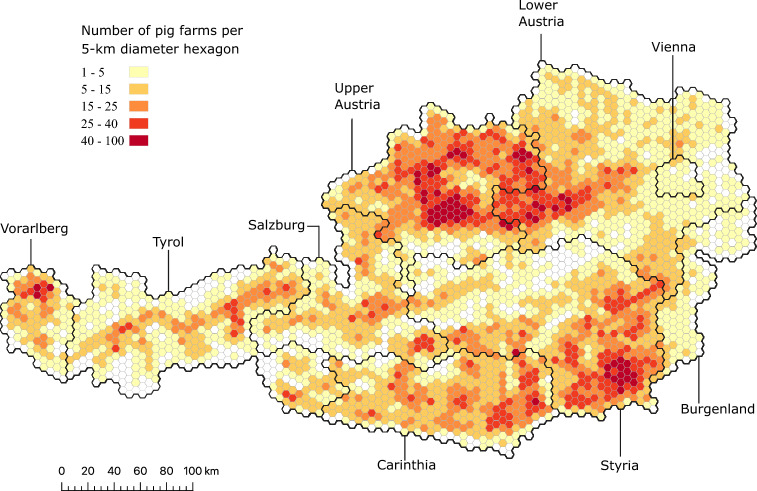
Spatial density of pig farms with recorded movements in Austria in 2021. The black lines represent the administrative borders of the federal states.

We observed that the spatial distribution of the Austrian pig farms remained stable over the study period 2015–2021 (Supplementary Video S1). Upper Austria showed the highest number and density of farms, counting on average for 25.3% (min. = 25.2%; max. = 25.5%) of the total number of Austrian farms every year, followed by Styria (mean = 20.6%; min. = 20.0%; max. = 20.1%). Figure 1 displays the spatial farm density for 2021. Overall, the network exhibited a high proportion of small-sized farms, with, on average, 62.9% (min. = 59.4%; max. = 65.5%) of the farms reporting four pigs or less per year over the study period. On the other hand, large-sized farms (i.e. farms presenting $\ge$ 2000 pigs) counted on average 0.25% (0.22–0.28%) of all farms. For example, in Upper Austria and Styria, the median size of the farms was two pigs per farm (Upper Austria: min. = 1; max. = 3045, Styria: 1–17,615).

**Figure 2:**
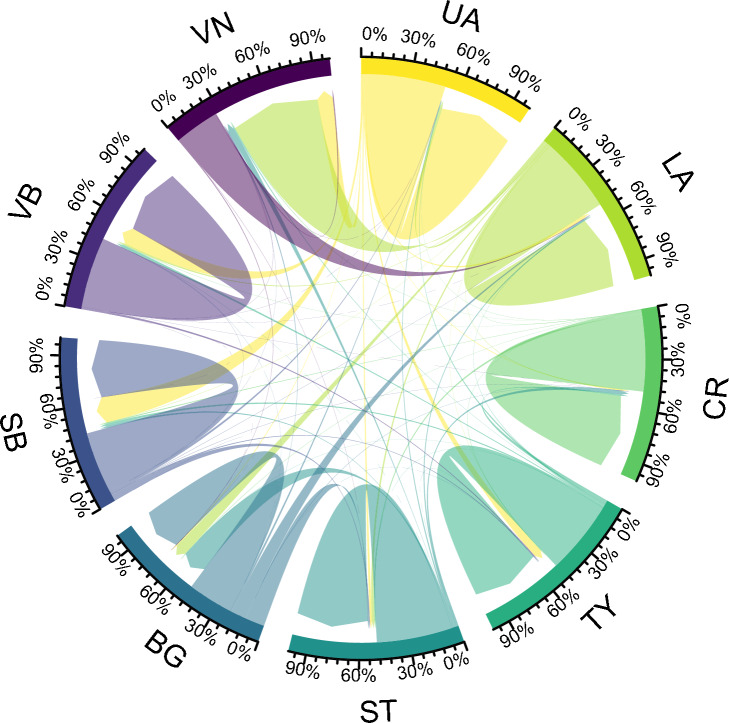
Edge bundling showing the proportion of intra- and inter-states pig movement flows in Austria, 2015–2021. Arrows’ weight represents the frequency of trades from one federal state to another. Arrows originating and ending in the same federal state indicate intra-state movements, while arrows pointing to other federal states show inter-state movements. BG: Burgenland; CR: Carinthia; LA: Lower Austria; SB: Salzburg; ST: Styria; TY: Tyrol; UA: Upper Austria; VN: Vienna; and VB; Vorarlberg.

Over the study period, the intra-state movements (i.e. trades that occurred between holdings located in the same federal state) were more frequent than inter-state movements (91.6% of all recorded movements versus 8.4%, respectively). The majority of the intra- and inter-state movements (41.7%) originated from Upper Austria, followed by Lower Austria (25.1%), Styria (17.8%), and Carinthia (8.0%). These four federal states exhibited the highest relative contribution to the total number of intra-state movements, representing 42.4%, 24.8%, 17.8%, and 8.3% of them, respectively (Fig. 2). In general, median batch size tended to be bigger for intra-state trades compared to inter-states movements (18 vs. 9, respectively).

### Yearly aggregated networks

Table 2 presents the network metrics for each yearly snapshot of the domestic pig movements. Results of the analyses that incorporated slaughter-type movements are reported in (Supplementary Results Table 1). Notably, both analyses resulted in similar metrics.

The degree assortativity (connection preference) of the yearly networks remained stable with a slight negative value over the seven years (mean = $-0.09 \pm \ 0.004$ (SD)). This means that holdings did not have any preference in connecting with other holdings based on the number of trades (degrees). Similarly, the (federal) state and district assortativity showed little yearly variations. However, both metrics exhibited positive values, demonstrating preferential (homophilic) trade relationships among pig holdings located in the same district (mean = 0.44 $\pm \ 0.010$) and even higher preference for pig holdings located in the same federal state (mean = 0.86 $\pm \ 0.012$), supporting observations from Fig. 2 which clearly illustrates that the majority of trades occurred within federal states.

The edge density displayed very low values (close to zero), which indicates the sparsity of connections between nodes in the network^3^. Similarly, the yearly global clustering coefficients were relatively small, varying from $3.9\times 10^{-3}$ to $5.4\times 10^{-3}$, showing low connectivity of each node with its neighbors. The network diameter and average shortest path length fluctuated annually but exhibited a decrease of 13.8% and 35.2%, respectively, over the study period. The network diameter showed relatively high values (min. = 19; max. = 29). The yearly average shortest path lengths showed medium values (min. = 6.2; max. = 10.7), meaning that any pair of holdings was distant by approximately 6–11 movements every year. These properties are only partially compatible with those of small-world networks, i.e. networks that show high clustering (like regular lattice) but have small characteristic path lengths (like random graphs)^1^. The network betweenness centrality was close to zero for each year. However, at node level, the betweenness score (min. = 4781; max. = 47,158) was highly skewed with 1% of all holdings showing betweenness scores $\ge$ 21,672, reflecting a greater capacity of these holdings to act as a bridge and spread an infectious pathogen across the network^5^.

**Figure 3:**
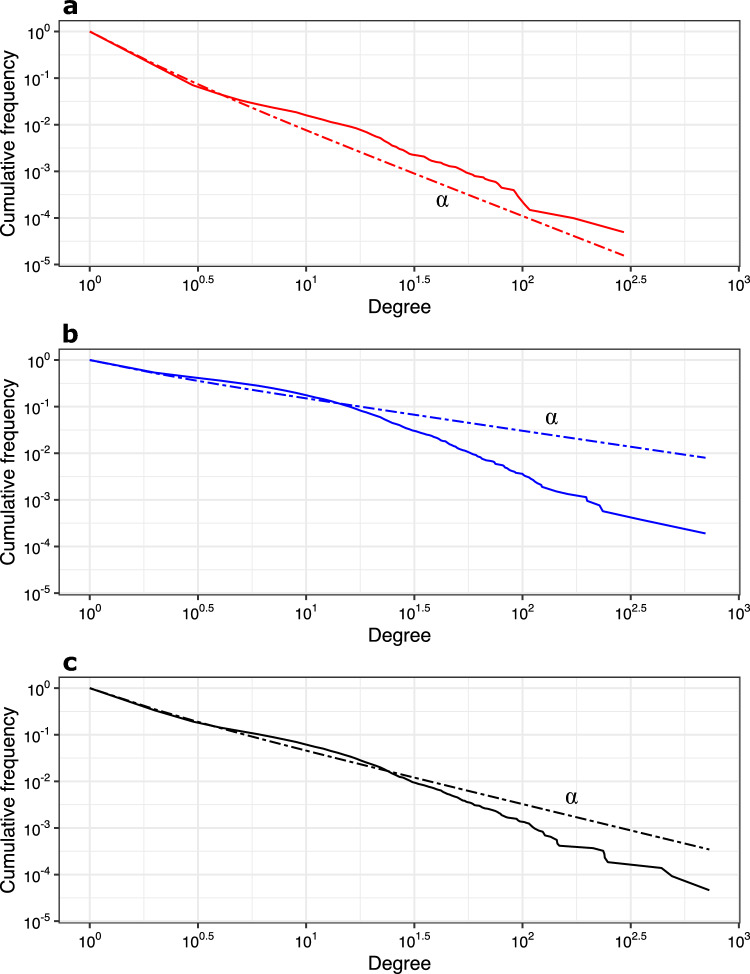
Degree distributions of the Austrian pig trade network for the year 2021. The cumulative frequencies of the node (**a**) in-degree, (**b**) out-degree, and (**c**) total degree distributions are represented on a log–log scale. Each degree distribution was approximated as a power law (dashed lines) using a maximum likelihood approach^97^, with $\alpha$, i.e. the law’s exponent, equals to 2.81, 1.68, and 2.12, respectively.

The yearly in-degree, out-degree, and total degree distributions were right skewed, heavy-tailed and could be approximated by a power law distribution with an average exponent of 2.79 $\pm \ 0.012$, 1.65 $\pm \ 0.017$, and 2.14 $\pm \ 0.008$, respectively (Supplementary Fig. S1). The degree distributions also showed that each year, the majority of nodes (on average 85%) had either out- or in- degree only. Figure 3 presents the results for 2021. The 1% most connected nodes counted yearly for 252 nodes on average (min. = 226; max. = 292), including 249 (223–288) pig farms on average. They counted on average for 0.22% (0.21–0.24 %) of all in-movements and 0.79% (0.76–0.81%) of all out-movements. In 2021, 74.3% of the top 1% highly connected nodes were similar to the top 1% highly connected nodes identified in 2015. Over the 7-year study period, these nodes were mostly located in Upper Austria, which represented on average 29% of all most connected nodes (min. = 27.3%; max. = 31.4%), followed by Styria (24.6%; 23.9–26%), Lower Austria (19.4%; 18.2–20.9%), and Carinthia (12.2%; 10.6–13.2%). We considered these top 1% of most connected nodes as *hubs* in the network. Among them, 35.7% (29.5–47.6%) were also identified as nodes with high betweenness. The typical topology of the Austrian pig trade network (i.e. sparse connectivity, skewed distribution of the network degrees, and rare *hubs*) is illustrated for Upper Austria (2021) in Supplementary Fig. S2.

For each year, the largest strongly connected component (LSCC) contained a very small fraction of the nodes (min. = 0.56; max. = 3.80%) (Table 2) whereas more than 95% of the nodes were included in the largest weakly connected component (LWCC). Among pig holdings included in the LSCC, 56% (min. = 49.5%; max. = 64.9%) were located in Upper Austria while the remaining ones were located in Styria (20.6%; 9.4–30%), Lower Austria (16.0%; 5.6–39.4%), and Carinthia (8.5%; 2.9–13.5%). Most of the nodes in the LSCC had farm label (92.2%; 85.1–99.5 %) and a small fraction presented other labels (combined or not with another label), such as trade (7.9%; 4.4–13.1%), collection point (4%; 2.6–6%), and abattoir (3.7%; 2–5%).

Computed from the yearly network snapshots, the node ranges^22^ in the Austrian pig trade network are displayed in Supplementary Fig. S3. Overall, nodes belonging to a LSCC exhibited longer ranges together with a broader range distribution (7299.71 $\pm \ 3529.6$ compared to other (non-LSCC member) nodes (363.93 $\pm \ 130.21$. Nodes not belonging to a LSCC showed both short and long ranges. The shortest range over all yearly snapshots was one, however, long-range nodes showed decreasing node range between 2015 (max. = 11,547) and 2021 (max. = 6593).

No cycle was detected in the network.

### Trends in the pig trade network

**Figure 4:**
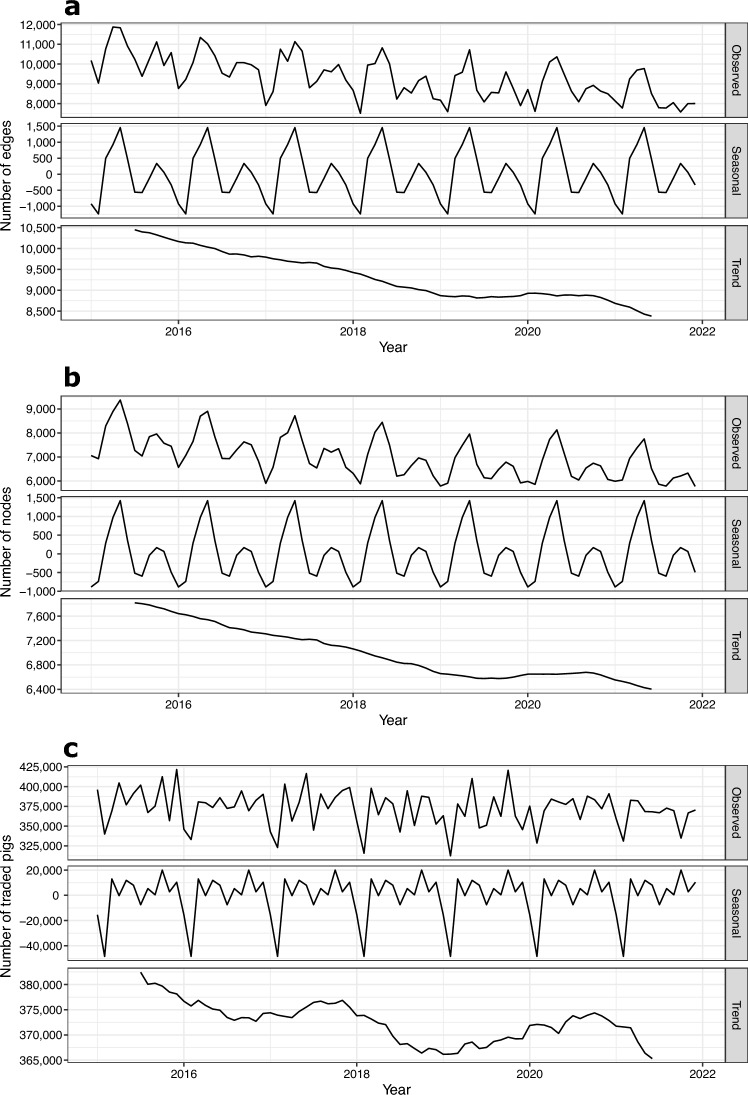
Classical time series decomposition with two-sided moving averages of the monthly number of (**a**) edges, (**b**) nodes with recorded movements, and (**c**) volume of traded pigs in Austria, 2015–2021.

The monthly time series of the number of nodes and edges showed a steadily decreasing trend from 2015 until the second semester 2019 followed by a slight uptrend until winter 2020, before both trends decreased again (Fig. 4a,b). Similarly, the monthly time series of the number of pig trades exhibited a negative trend over the study period, showing, however, downward (2015–2016, 2018, first semester 2021) and upward (2017, 2019–2021) short-term trends (Fig. 4c).

The monthly number of edges and nodes showed marked and correlated seasonal bimodal patterns over the period 2015–2021, with a first peak in May and a second one in October, whereas January-February and July–August exhibited the lowest node and edge activities. On the contrary, the volume of traded pigs showed little monthly variations except a seasonal sharp decrease in February (Fig. 4, Supplementary Fig. S4). Furthermore, at a more granular level, the weekly time series of nodes, edges, and volume of traded pigs, exhibited a strong week-end effect, with a growing activity from Monday to Friday and a lower activity on Saturday and Sunday. The weekly time series of the movements of animals intended for slaughter (excluding other types of movement) exhibited an opposite weekly pattern (Supplementary Figs. S5–S7).

The average daily number of nodes, edges, and volume of traded pigs did not show any significant difference between months neither for domestic (*p*-*value* = 0.33, 0.25, 0.86, respectively)nor slaughter-type movements (*p*-*values* = 1).

### Dynamic community detection

**Figure 5:**
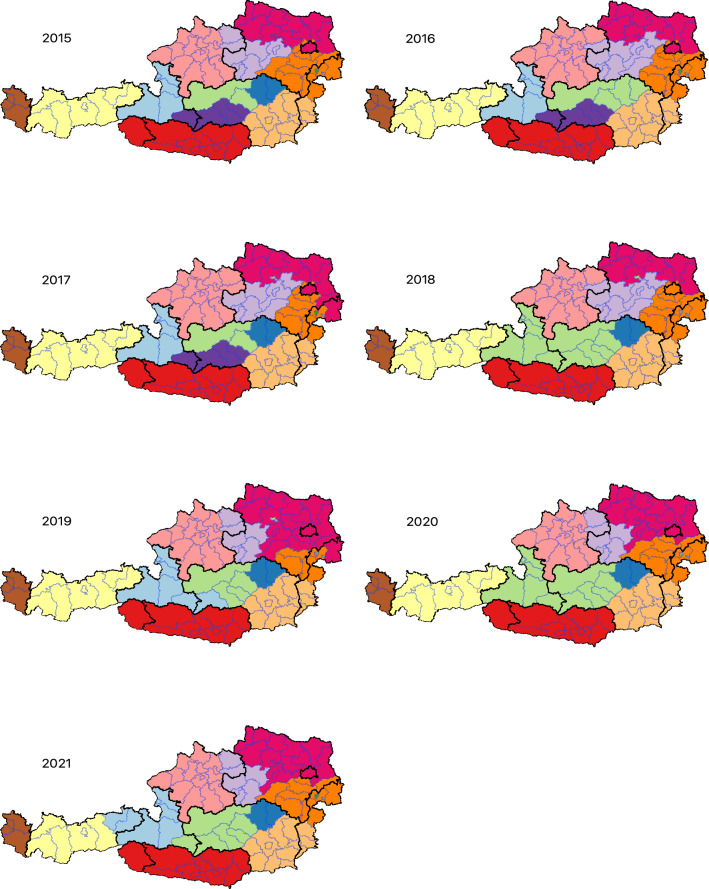
Maps of the detected trade community based on the yearly aggregated networks of pig movements in Austria, 2015–2021. Blue lines represent the district administrative boundaries; black dashed lines represent federal state boundaries. Colors represent communities. We used the InfoMap algorithm^67^ that allows no overlap, i.e. a district can belongs to one community only.

The number of trade communities in the Austrian pig movement network showed little variations among the yearly snapshots, with 10–13 communities detected annually (Fig. 5). The median trade community size was also relatively stable, varying between six and nine districts per community (min. = 1; max. = 19). The pairwise comparison of the community topology between year 2015 (reference) and each other subsequent year showed that the trade community structure did not change significantly during the seven years. This result was supported by the year-over-year comparison of the trade community structure (Table 3). The Kruskal-Wallis test was used to compare the average matching values in both series (2015 as reference and year-over-year comparison) and confirmed that there were no significant difference over the study period (*p*-*value* = 0.68 and 0.63, respectively).

As observed in Fig. 5, a trade community may consist of districts from different federal states (e.g. black dashed-line representing the federal state boundaries on top of the purple colored community in 2015). While some communities divided some federal states, others bridged over two of them. Moreover, some communities have a very dynamic behavior, i.e. a community may “loose” some member districts or “merge” partially with another community. For example, the three central communities detected in 2017 (colored in blue, purple, and green) formed one single community in 2018 (green), which then split into two communities (2019) and latter re-assembled (2020).

## Discussion

This study aimed to explore, for the first time, the network of pig movements in Austria using methods derived from social network analysis and reveal its dynamic structure. Below, we provide an epidemiological perspective on this network, which suggests alternative, network-based applications to the monitoring and management of infectious diseases.

We evidenced that the spatial distribution of the pig farms in Austria was heterogeneous, with two federal states, Upper Austria and Styria, accounting for almost half (46%) of all farms with trading activity in the country, whereas the pig farming system was largely dominated by small-sized farms of four pigs or less. Spatial heterogeneity influences the size and duration of an infectious disease outbreak^73^. Typically, the risk of farm-to-farm transmission increases with the farm density (independently of livestock movements) whereas farms with higher animal density are more susceptible to outbreaks^74,75^. Additionally, our findings showed that the Austrian pig production was shifting toward fewer holdings and trades, while the volume of animals produced did not exhibit a correlated decrease, which indicates that the farm and batch size are becoming larger. Although this information looks trivial, knowing precisely the spatial distribution of farms and animals is necessary to assess the potential impact of interventions and plan resource allocation for surveillance and control of infectious diseases^76^. It is also important to understand how shifting production patterns toward intensification (i.e. fewer but larger farms) may influence disease risk on a long term.

The topology of the pig trade network, unfolded in the yearly aggregated snapshots, suggested sparse connectivity, along with a great proportion of nodes exhibiting either an in- or out-degree equal to zero, and the absence of cycle. The topology also revealed the small size of the yearly LSCC. These findings reflect the typical pyramidal pig farming system in Austria, with its tree-like, hierarchical structure and unidirectional animal flow, where batches of pigs move sequentially from a compartment (i.e. production stage) to the next one, till the end of the production chain (i.e. from farrowing to finishing and then to slaughterhouse). The pyramidal pig farming system can also be found in other countries, e.g. in Denmark^20^, France^24^, and the United States^62^. Furthermore, we proved that holdings showed a strong tendency for tradings with other holdings located in the same federal state whereas a smaller proportion of movements covered long-distances.

The yearly patterns of pig movements in Austria showed the topology of a scale-free network, with a large heterogeneity in the node degrees (i.e. number of connections for each pig holding), unveiling the presence of few highly connected nodes (*hubs*). Holdings with a high out-degree and/or high betweenness can act as super-spreaders and should be targeted with priority i) in disease surveillance programs (farm sentinels) in order to achieve cost-effective and efficient early disease detection, and ii) in control interventions (e.g. node removal to increase the fragmentation of the network and minimize the number of exposed holdings)^16,20,77–80^. From an epidemiological perspective, the in-degree and betweenness of a holding indicate its vulnerability to infection (from another holding) and pig holdings showing a high in-degree can be considered as *super-receivers*. On the other hand, the out-degree of a node reflects its capacity to spread pathogens. Enhancing biosecurity in highly connected premises (*hubs*), that act as “gatekeepers”, would greatly reduce the structural risk and favor timely detection of pathogens (early warning)^38,81^. These findings would certainly need further empirical verification through field testing, especially to assess their practical applicability and real-world implications.

The domestic swine holdings of Upper Austria, Styria, and eastern Lower Austria showed a higher connectivity (i.e. greater relative proportion of holdings belonging to the LSCC) and longer node ranges. We demonstrated that the LSCC, which is a standard proxy to assess the lower bound of an outbreak final size^12^, was distributed over several federal states, which may increase the risk of multi-state outbreak^6^. These findings indicate a higher structural vulnerability of Upper Austria, Styria, and eastern Lower Austria to infectious disease outbreaks, compared to other federal states. Reinforcing surveillance and biosecurity in pig holdings located in these high-risk regions will probably limit epidemic spread and reduce outbreak sizes.

Overall, the absence of small-world like structure, the low average degree of the nodes, and the rare long-range movements favor the network resilience to epidemics^82^ and supports the hypothesis that, at national scale, the size of an infectious disease outbreak in the Austrian pork production chain would be limited^81,83^. This statement is supported by the relatively small size of the LSCC and the relatively high diameter of the network (e.g., the reported diameter of the German pig trade network was 18^22^).

The analysis of seven successive years of pig movement data underlined recurrent patterns and seasonal behaviors in trade activities. Especially, our results confirmed a greater activity in spring (during the Easter holidays) and autumn (before Christmas) whereas the lowest activity occurred in winter, consistent with other European countries^33,79,84^. Temperatures play a role on animal health in general, as well as on the maintenance, seasonal pattern, and transmission of infectious pathogens, especially respiratory pathogens^85,86^, within livestock trade networks^75,87^. Seasonal patterns in the network activities, linked with the swine breeding and production cycle, consumer demand as well as climatic conditions should be considered when planning surveillance activities and responding to an outbreak.

The trade communities covered geographic areas of various sizes and their limits did not correspond to the federal state administrative boundaries. Trade communities reflect connection preferences between pig holdings, i.e. pig production zones. Community structure greatly influences disease dynamics^88^ and represents a useful epidemiological tool to delineate compartments or zones that could be used in preventive veterinary medicine, e.g. to design geographically-targeted surveillance strategies as well as control and eradication programs^42,89^. The implementation of a community-based approach to disease control (i.e. not based on administrative boundaries) would preserve commercial exchanges, have a lower impact on the pig production chain, and therefore would probably be cost-effective. We argue that disease management at sub-national level can be relevant, yet this compartmentalization approach should be further investigated. Indeed, the InfoMap algorithm generates disjoint communities, i.e. in our case, a district belongs to one and only one community^67^. Yet in real-world networks, communities have important overlapping and may present nested structures^90,91^. Detecting overlapping districts may unveil “bridges” between pig trade communities, which may be crucial for both epizootic and endemic disease control efforts.

The yearly structure of the Austrian pig trade network as well as its communities were rather stable over the seven-year study period. These results support the use of retrospective movement data, as well as previously established analytical frameworks and results, to quickly respond to emergency situations that may occur in the network (e.g. infectious disease outbreak or disaster). The network stability and specific tree-like structure present also a major advantage for planning a simple, network-based targeted surveillance strategy, e.g. by using a static network analysis approach^22^. Nonetheless, estimation of the outbreak size requires a time-resolved analysis, where the time window for data aggregation largely depends on the dynamics of the network (e.g. seasonality) and characteristics of the disease (e.g. incubation period, $R_0$)^92^, especially in the early phase of an epidemic^11^. In this study, we described a network which edges are weighted based on the trade frequency. In the future, it will be interesting to compare it with a network where edge weights refer to batch size and study how this influences the risk of infectious disease introduction in farms and propagation in the network.

Although comparing pig trade network properties between countries is challenging due to, e.g. differences in the period covered by the data, algorithms used, country size, and economic considerations, evaluating disparities and similarities in national pig trade structure and patterns can help identifying countries that may be more vulnerable to disease introduction^93^, while contextualizing our results within a European framework. Surprisingly, the Austrian pig movement network did not exhibit a small-world effect, which is usually observed in livestock networks presenting a lot of large commercial farms, such as in Germany, France, or Denmark, which also exhibit a denser network and, on average, higher degree centrality^21,22,24,93^. In contrast, the Austrian pig trade network showed similarities with the North Macedonian, Georgian, and Bulgarian pig movement networks, which are similarly weakly connected and involve a majority of small-scale holdings^93–95^. Also, the proportion of nodes included in the LSCC of the Austrian pig trade network was comparable to the one reported for the North Macedonian network (1.03–4.08%)^95^, but larger than the proportion reported for Bulgaria (0.15%), France (0.60%), Italy (0.14%), and Spain (0.18%)^93^. However, its density was closer to the density reported for the French network ($6.1 \times 10^{-5}$) whereas the shortest path length was closer to the one reported from Italy (11.2)^93^.

Our analysis has several limitations. First, the geo-coordinates of the holdings were randomized within a 5 km radius, which could have resulted in erroneous assignment of holdings located near administrative boundaries to a nearby federal state or district during the state/district attribution process in QGIS. However, we believe this had a very limited impact on our results as the number of affected holdings is small. Second, because the reporting is mandatory, we assumed that there was no missing data. Therefore, holdings found with no movement record during the entire study period or part of it may be small farms producing pigs for self-consumption or holdings which activity status in the system is outdated (this type of error cannot be tracked). Third, we found that pig movements in Austria were dominated ( 60%) by movements intended for slaughter, which we did not include in the main analysis because we considered that they have a limited impact on infectious disease spread^21^. This is true when pigs from one farm are sent directly to the slaughterhouse in a clean and disinfected vehicle. While we have assumed that the data accurately reflected the pig trade patterns, it is important to acknowledge that truck sharing is common, which may impact the network topology, e.g. by increasing its connectivity^83,96^ and modifying its community structure. Moreover, truck contamination enhances the risk of disease propagation; this is especially true for diseases that can survive outside of an animal and spread through fomites (e.g. FMD, ASF), unless very strict biosecurity measures at farm gate are enforced^69^. Transport itinerary data is not available in the database of the Veterinary Authorities and is therefore not used for epidemic management and control. For this reason, we believe our analysis reflects the best possible outcome that could be achieved with the available data. Certainly, the additional use of transportation itinerary data (i.e. truck movements) could improved or refined the analysis of this network and would advance our understanding of the risk of disease spread via direct (direct trade) and indirect (truck sharing) contact between holdings.

Overall, this study advances our understanding of the complex structure of the pig trade network in Austria and provides useful epidemiological information to designing cost-effective infectious disease control plans. Our findings highlight that when designing epidemiological surveillance activities and implementing disease control measures in the pig trade network in Austria, several components should be considered: the presence of high-farm density and highly-connected pig holdings, the seasonality of the network, and the presence of communities.

## Supplementary Information

Supplementary Information 1.

Supplementary Information 2.

Supplementary Information 3.

Supplementary Information 4.

Supplementary Information 5.

Supplementary Information 6.

Supplementary Information 7.

Supplementary Information 8.

Supplementary Information 9.

## Data Availability

The metadata and R code used to produce the results of this study are publicly available in Figshare at: 10.6084/m9.figshare.21904995.v1. The raw data that support the findings of this study are available from the Austrian Federal Ministry of Social Affairs, Health, Care and Consumer Protection (BMSGPK) but restrictions apply to the availability of these data, which were used under license for the current study, and so are not publicly available. Data are however available from the authors upon reasonable request and with permission of data owner.
