# Supplementary material for: Network analysis of pig movement data as an epidemiological tool: an Austrian case study
Source: Sci Rep. 2023 Jun 14;13:9623. doi: 10.1038/s41598-023-36596-1 (PMC10267221; doi:10.1038/s41598-023-36596-1)
Supplement: Supplementary file 1 — Supplementary Information 1. [file 41598_2023_36596_MOESM1_ESM.pdf]

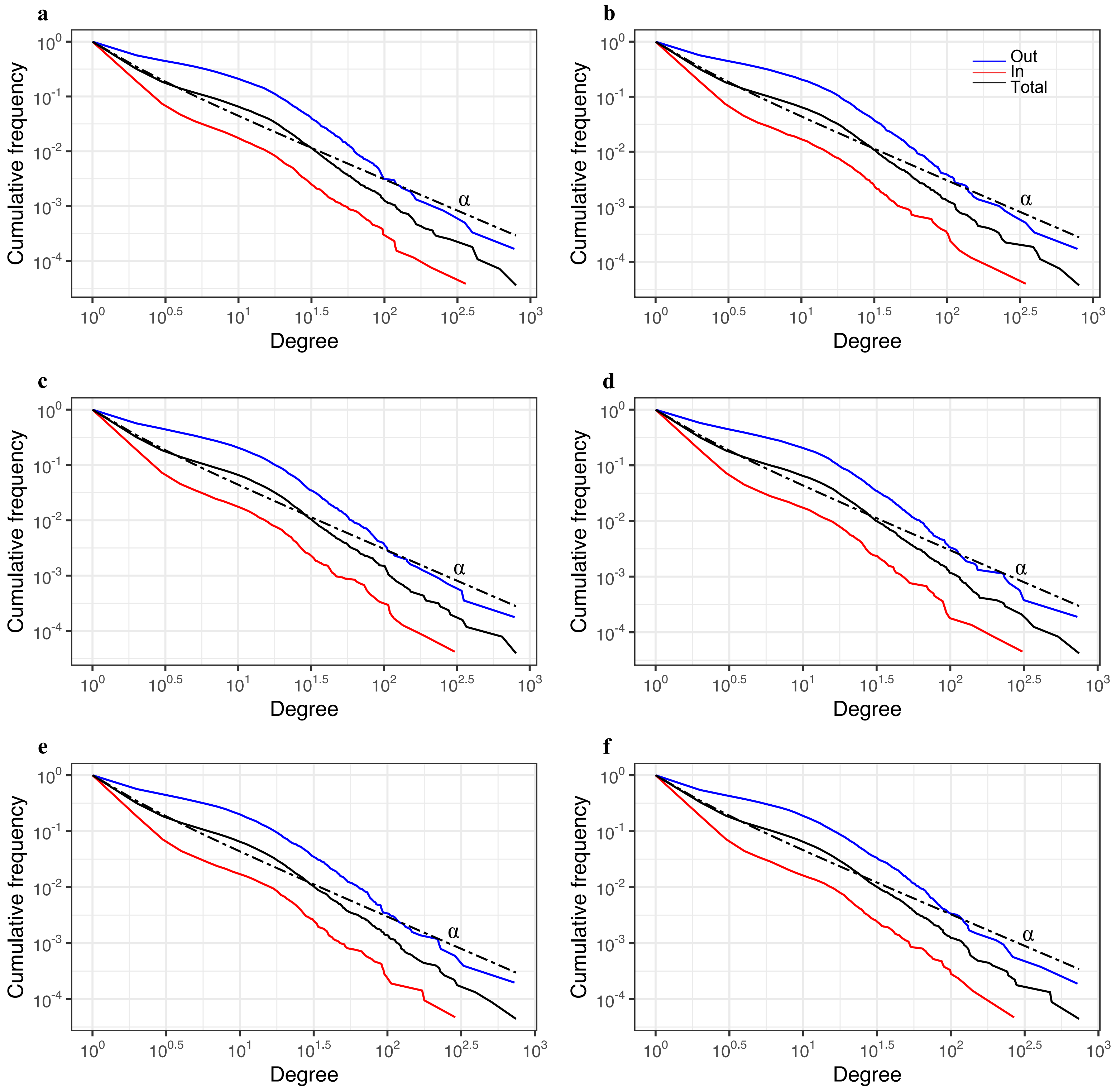

Supplementary Figure S1. Degree distributions of the Austrian pig trade network, 2015-2020. The cumulative frequencies of the node in-degree (red), out-degree (blue), and total-degree (black) distribution are represented on a log-log scale. Each degree distribution was approximated by a power law using a maximum likelihood approach. The signature of the power law is shown for the total degree distribution only. The values of the power law exponents (α) for the in-, out-, and total- degree distribution were very similar among years and equal to **a)** 2.77, 1.63, and 2.14, respectively, in 2015; **b)** 2.78, 1.64, and 2.14, respectively, in 2016; **c)** 2.78, 1.64, and 2.14, respectively, in 2017; **d)** 2.79, 1.64, and 2.14, respectively, in 2018; **e)** 2.81, 1.65, and 2.14, respectively, in 2019; **f)** 2.79, 1.67, and 2.12, respectively, in 2020.
