# Supplementary material for: Network analysis of pig movement data as an epidemiological tool: an Austrian case study
Source: Sci Rep. 2023 Jun 14;13:9623. doi: 10.1038/s41598-023-36596-1 (PMC10267221; doi:10.1038/s41598-023-36596-1)
Supplement: Supplementary file 2 — Supplementary Information 2. [file 41598_2023_36596_MOESM2_ESM.pdf]

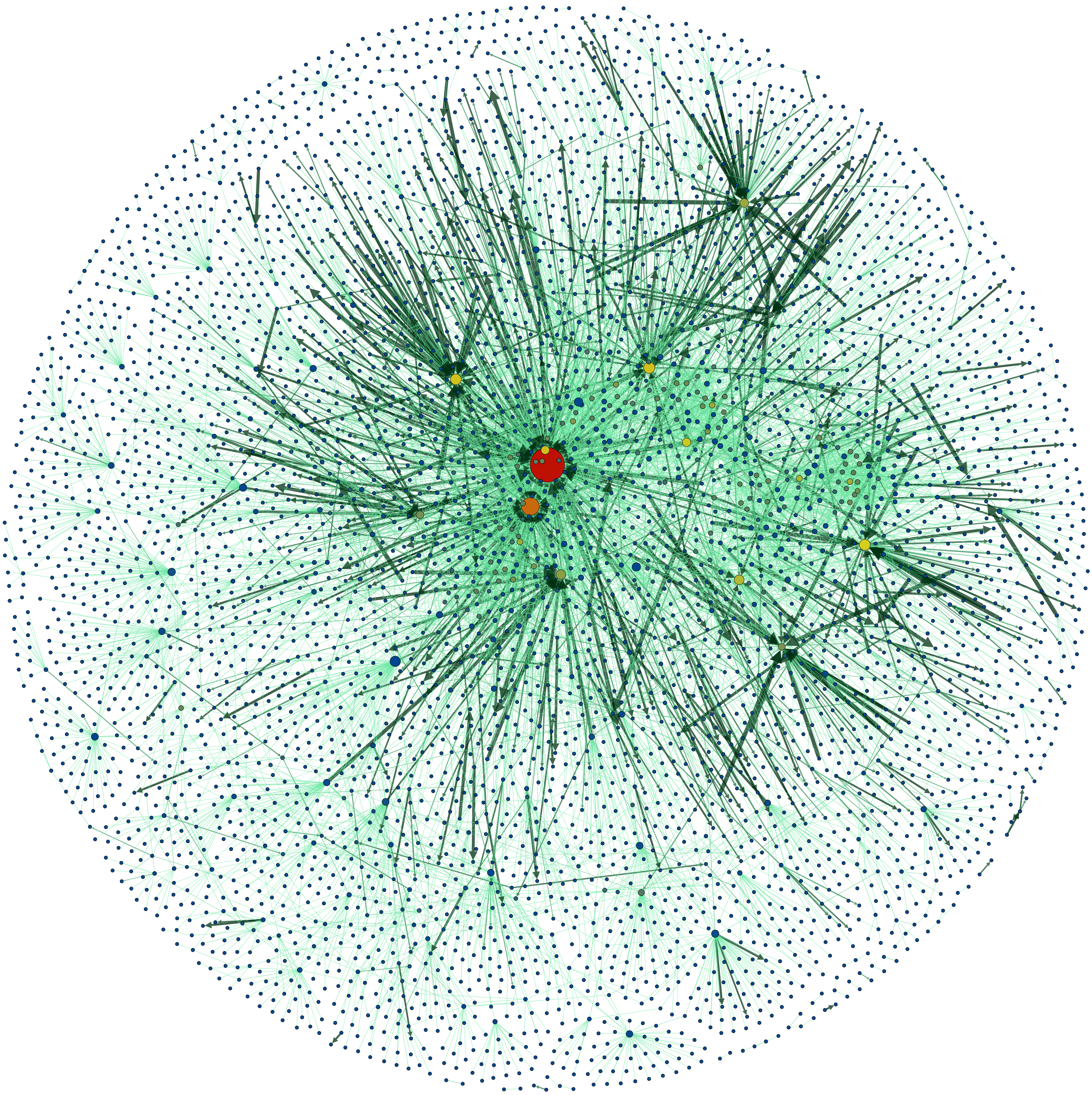

Supplementary Figure S2. Visualization of the directed network of pig movements in Upper Austria in 2021. Isolated nodes are not shown. The node size corresponds to the total degree. The node gradient color corresponds to the in-degree, from blue (low in-degree) to red (high in-degree). Arrows indicate the direction of the trades. The edge width corresponds to the trade frequency, i.e. the wider the edge the higher the trading frequency between a pair of nodes. The graph highlights the pyramidal structure of the pig farming system in Austria, showing a tree-like structure.
