# Supplementary material for: Network analysis of pig movement data as an epidemiological tool: an Austrian case study
Source: Sci Rep. 2023 Jun 14;13:9623. doi: 10.1038/s41598-023-36596-1 (PMC10267221; doi:10.1038/s41598-023-36596-1)
Supplement: Supplementary file 3 — Supplementary Information 3. [file 41598_2023_36596_MOESM3_ESM.pdf]

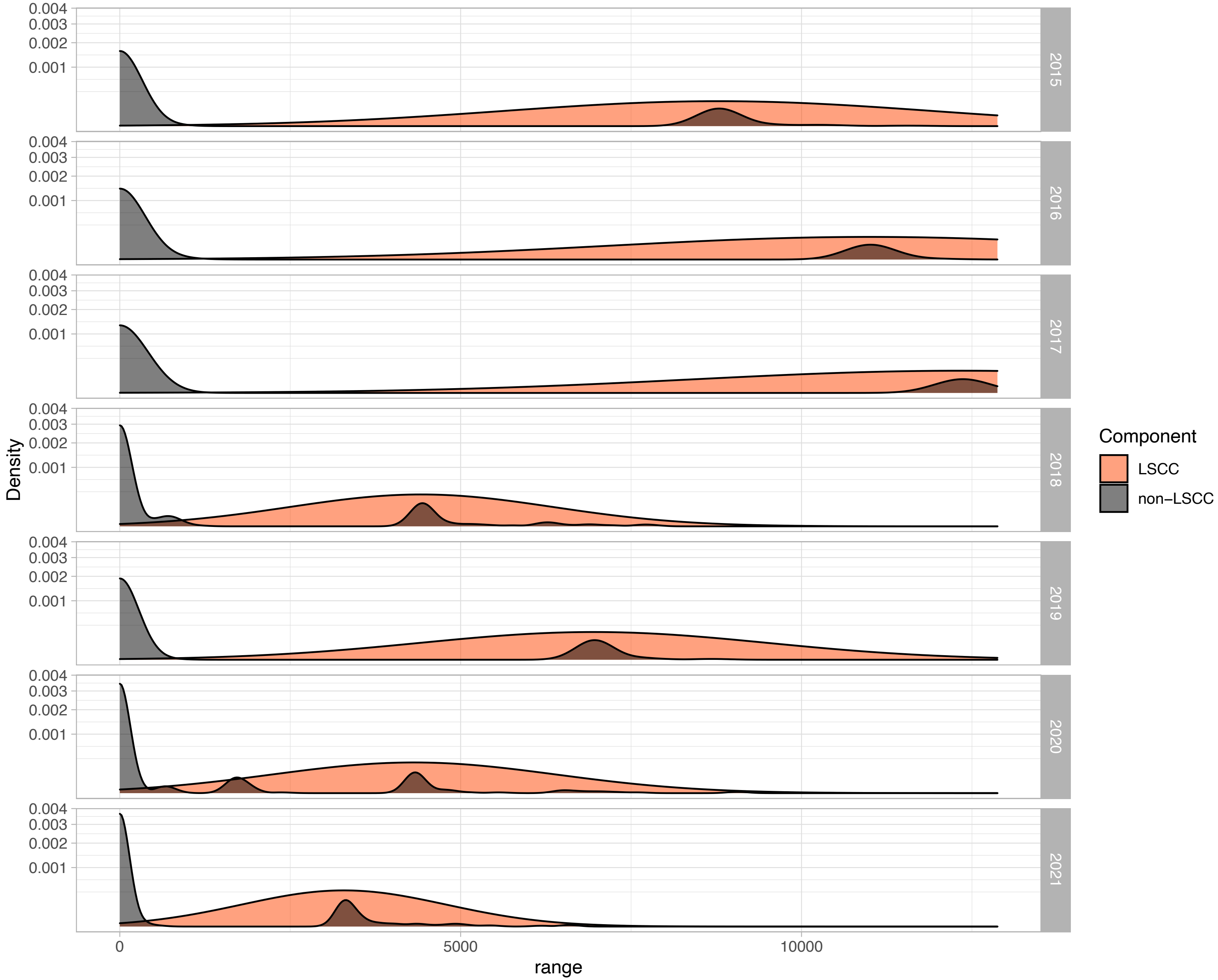

Supplementary Figure S3. Density plots of the yearly nodes' range distribution of the Austrian pig trade network, 2015-2021. Data are stratified by node membership, i.e. member of the largest strongly connected component (LSCC) versus non-LSCC members. A range is the number of nodes that can be reached from node *i* through a path of random length (Lentz *et al.*, 2013). The y axis (density) is transformed into square root scale to enhance readability.
