# Supplementary material for: Network analysis of pig movement data as an epidemiological tool: an Austrian case study
Source: Sci Rep. 2023 Jun 14;13:9623. doi: 10.1038/s41598-023-36596-1 (PMC10267221; doi:10.1038/s41598-023-36596-1)
Supplement: Supplementary file 4 — Supplementary Information 4. [file 41598_2023_36596_MOESM4_ESM.pdf]

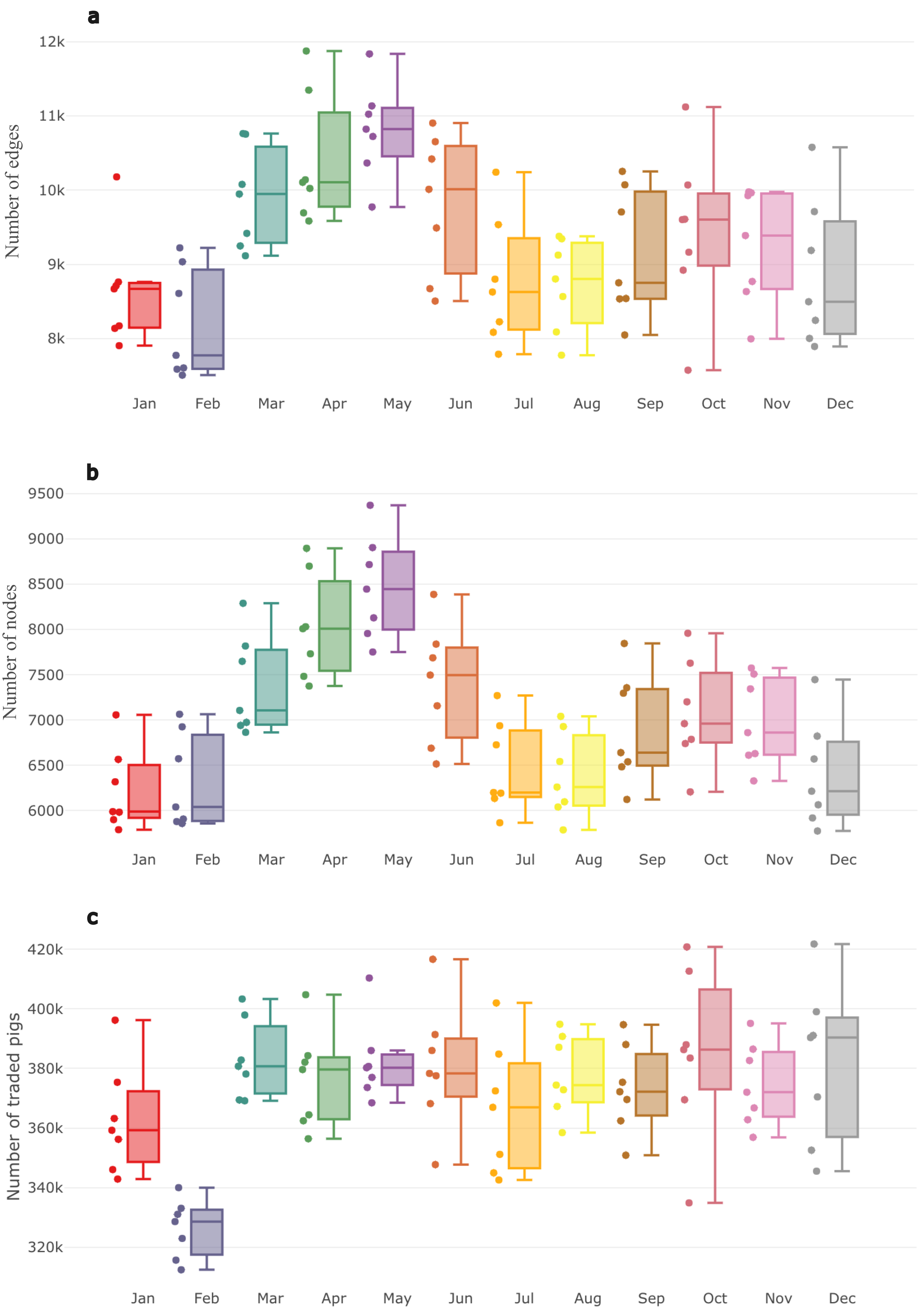

Supplementary Figure S4. Boxplots of the monthly number of (a) active edges, (b) active nodes, and (c) volume of traded pigs in Austria, 2015-2021.
