# Supplementary material for: Network analysis of pig movement data as an epidemiological tool: an Austrian case study
Source: Sci Rep. 2023 Jun 14;13:9623. doi: 10.1038/s41598-023-36596-1 (PMC10267221; doi:10.1038/s41598-023-36596-1)
Supplement: Supplementary file 6 — Supplementary Information 6. [file 41598_2023_36596_MOESM6_ESM.pdf]

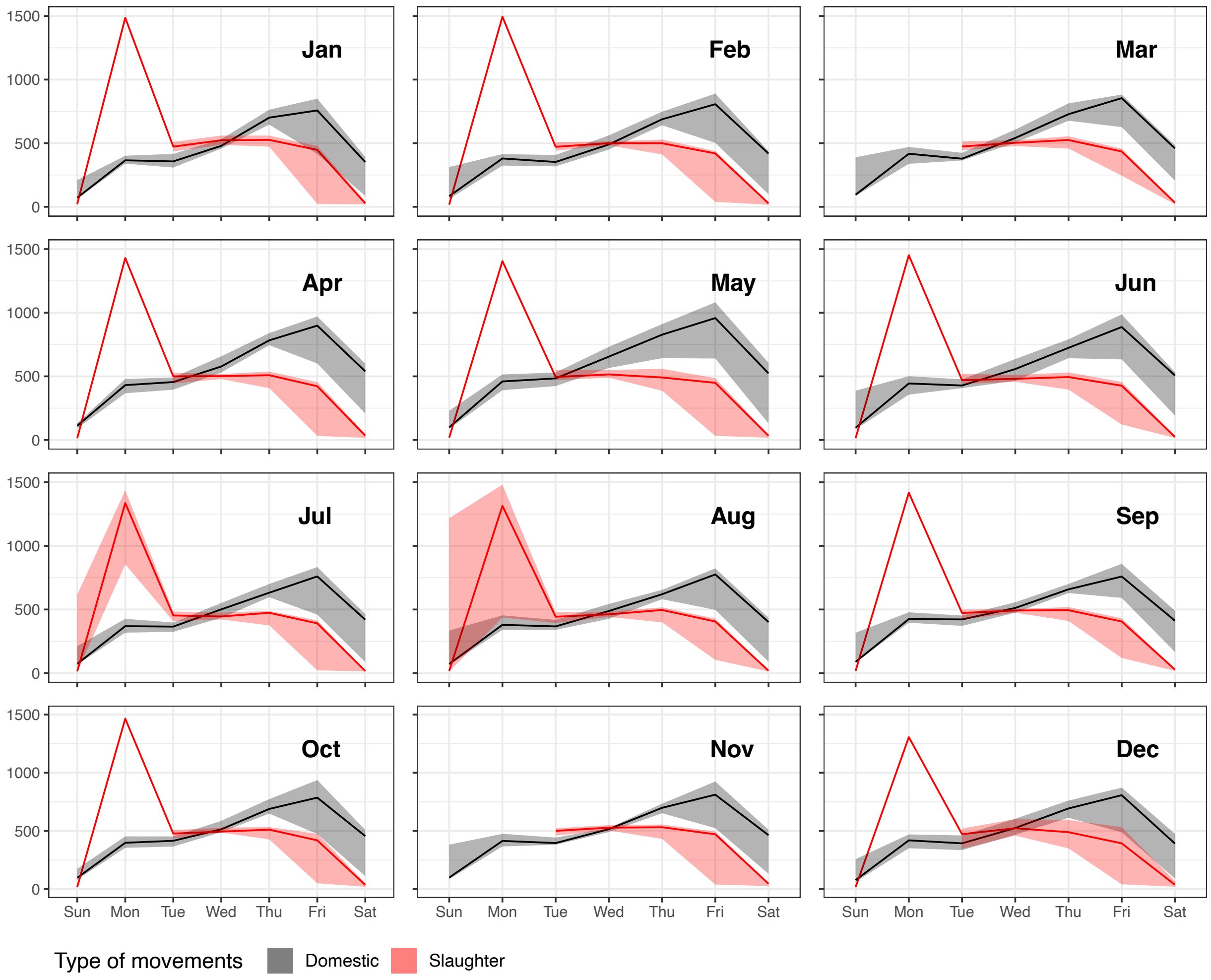


**Supplementary Figure S6.** Density plots of the number of nodes (pig holdings) for domestic- and slaughter- type movements in the Austrian pig trade network aggregated to weekdays, 2015-2021. Each graph represents a month. Lines represent the median monthly values computed for the seven-year study period while shaded areas represent the minimum and maximum number of nodes per month. Friday exhibited the highest activity regarding domestic pig movements (when depicted by the number of holdings) in all months. While movements intended for slaughter peaked on Monday. An analysis of variance (ANOVA) revealed no significant difference in the weekday activity among months for both domestic and slaughter movements, *p-values* of 0.34 and 1, respectively.
