# Supplementary material for: Network analysis of pig movement data as an epidemiological tool: an Austrian case study
Source: Sci Rep. 2023 Jun 14;13:9623. doi: 10.1038/s41598-023-36596-1 (PMC10267221; doi:10.1038/s41598-023-36596-1)
Supplement: Supplementary file 8 — Supplementary Information 8. [file 41598_2023_36596_MOESM8_ESM.pdf]

Supplementary Results

These Supplementary Results provide an additional network analysis that includes both domestic- and slaughter-type movements. The methods used are described in the Methods section of the main article.

Yearly-aggregated networks

Supplementary Table 1 presents the metrics of the yearly-aggregated networks (see Methods in the main article).

Dynamic community detection

Supplementary Figure S8 presents the outputs of the community detection performed at district level using the InfoMap, and for each yearly-aggregated network.

The number of trade communities showed very little variations among the yearly snapshots, with 10 to 11 communities detected annually. The median trade community size was relatively stable, varying between eight and nine districts per community (min. = 2; max. = 20).

We calculated the matching values to compare each community between consecutive years (year-over-year comparison) and to compare each community detected in the first snapshot (2015), used as a reference, with the subsequent ones (2016 to 2021), i.e. 2015 vs. 2016, 2015 vs. 2017, etc. Moreover, by averaging the matching values over all communities, we calculated a global matching value in every time window to observe the overall stability of the communities in the Austrian pig trade network over the study period (see Methods for more details). Results are reported in Supplementary Table 2.

The Kruskal-Wallis tests indicated that there were differences in the average matching values between the yearly snapshots (both tests provided a $p-values = 0.01$). We then used a Conover test to perform post hoc pairwise multiple comparisons. Results showed that, when using a reference snapshot (2015), communities were statistically different between 2015 and 2018 as well as between 2015 and 2019, with $p-values$ of 0.03 and 0.006, respectively. The year-over-year comparison did not show significant differences in the communities.

	2015	2016	2017	2018	2019	2020	2021
Number of edges	298,677	287,684	275,691	265,757	257,324	254,510	245,431
Number of nodes	30,338	29,154	27,592	26,183	24,860	24,565	23,702
Number of animals	9,505,308	9,335,911	9,291,499	9,163,590	9,033,015	9,131,562	9,004,316
Number of farms	29,763	28,532	27,003	25,908	24,180	24,038	23,197
Assortativity (degree)	-0.17	-0.17	-0.16	-0.17	-0.17	-0.17	-0.16
Assortativity (state)	0.85	0.84	0.84	0.84	0.83	0.82	0.82
Assortativity (district)	0.42	0.42	0.42	0.41	0.41	0.40	0.40
Average shortest path length	30.1	24.7	30.5	24.1	21.4	29.5	22.6
Betweenness centrality	0.01	0.01	0.03	0.01	0.02	0.01	0.01
Diameter	29	29	26	29	25	28	25
Degree (total)	0.02	0.02	0.02	0.02	0.02	0.02	0.02
Degree (in-)	0.04	0.03	0.03	0.04	0.04	0.04	0.04
Degree (out-)	0.03	0.03	0.03	0.03	0.03	0.03	0.03
Edge density	7.5×10^{-5}	7.7×10^{-5}	8.1×10^{-5}	8.6×10^{-5}	9.2×10^{-5}	9.3×10^{-5}	9.5×10^{-5}
Global CC	6.7×10^{-3}	6.5×10^{-3}	6.7×10^{-3}	6.7×10^{-3}	6.7×10^{-3}	6.1×10^{-3}	6.3×10^{-3}
Size LSCC (No. nodes)	685	932	1369	535	844	301	224
Size LSCC (% of nodes)	2.3%	3.2%	5.0%	2.0%	3.4%	1.2%	0.9%
Size LWCC (No. nodes)	30,140	28,995	27,345	25,997	24,703	24,402	23,536
Size LWCC (% of nodes)	99.3%	99.5%	99.1%	99.3%	99.4%	99.3%	99.3%

Supplementary Table 1. Summary of the graph-level metrics for the yearly snapshots of the Austrian pig trade network, which includes both domestic- and slaughter- type movements, covering from the period of 2015 to 2021. CC: clustering coefficient; LSCC: largest strongly connected component; LWCC: largest weakly connected component.

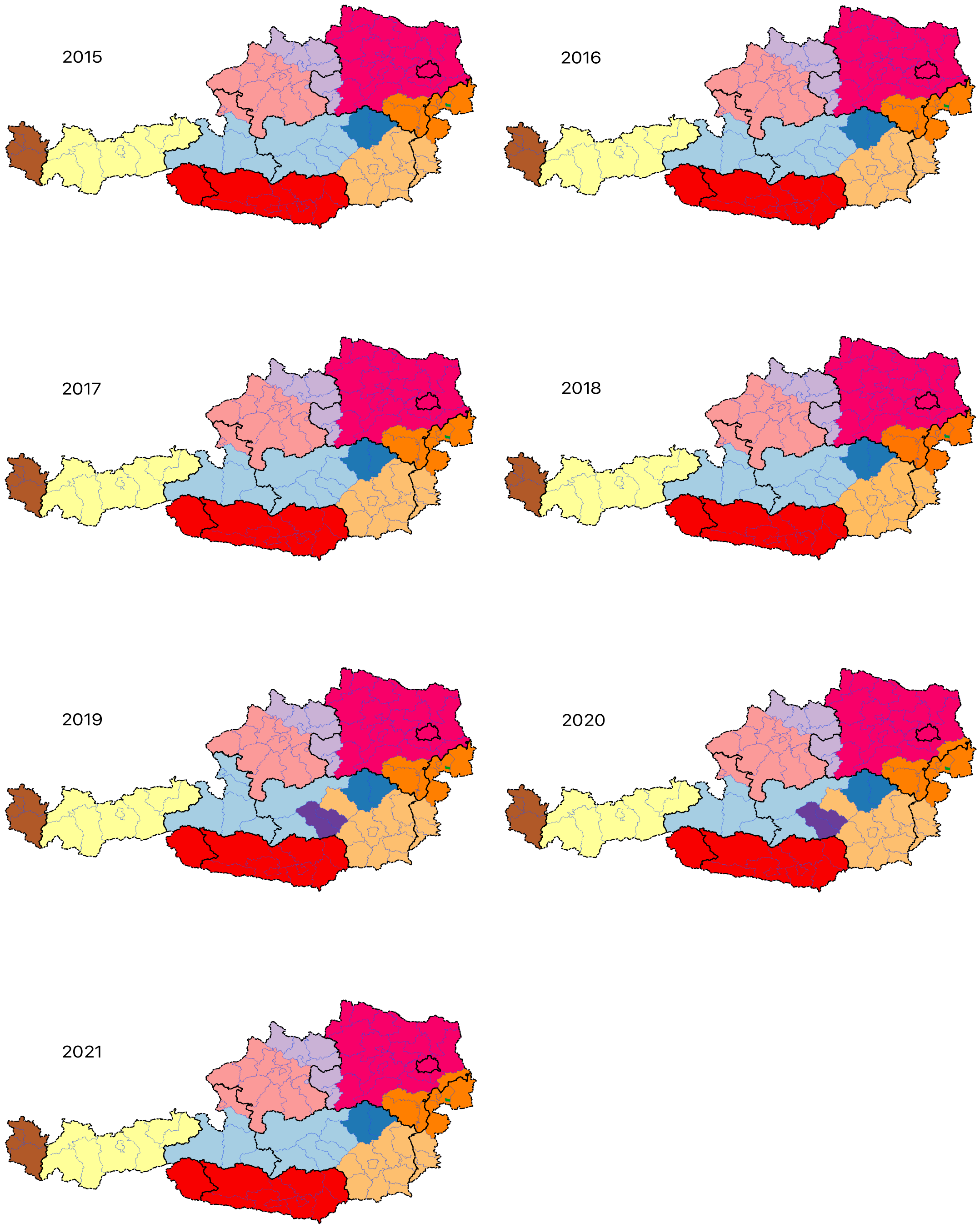

Supplementary Figure S8. Maps of the detected trade community based on the yearly aggregated networks of pig movements in Austria, 2015-2021, including domestic- and slaughter-type movements. Blue lines represent the district administrative boundaries; black dashed lines represent federal state boundaries. Colors represent communities. We used the InfoMap algorithm[?] that allows no overlap, i.e. a district can belongs to one community only.

Comparison	Average community matching value ± SD
**2015 (as reference)**	
2015 - 2016	1
2015 - 2017	0.9 ± 0.28
2015 - 2018	0.9 ± 0.28
2015 - 2019	0.7 ± 0.39
2015 - 2020	0.69 ± 0.4
2015 - 2021	0.9 ± 0.28
**Year-over-year**	
2015 - 2016	1
2016 - 2017	0.9 ± 0.28
2017 - 2018	1
2018 - 2019	0.76 ± 0.37
2019 - 2020	0.82 ± 0.33
2020 - 2021	0.75 ± 0.37

Supplementary Table 2. Average community matching values (SD: standard deviation) compare communities detected in 2015 (as reference) based on slaughter movements with those in each subsequent year of the Austrian pig trade network. Further, the year-over-year average community matching values, 2015-2021, were also computed.
