# Supplementary figures and images for: Network analysis of pig movement data as an epidemiological tool: an Austrian case study
Source: Sci Rep. 2023 Jun 14;13:9623. doi: 10.1038/s41598-023-36596-1 (PMC10267221; doi:10.1038/s41598-023-36596-1)

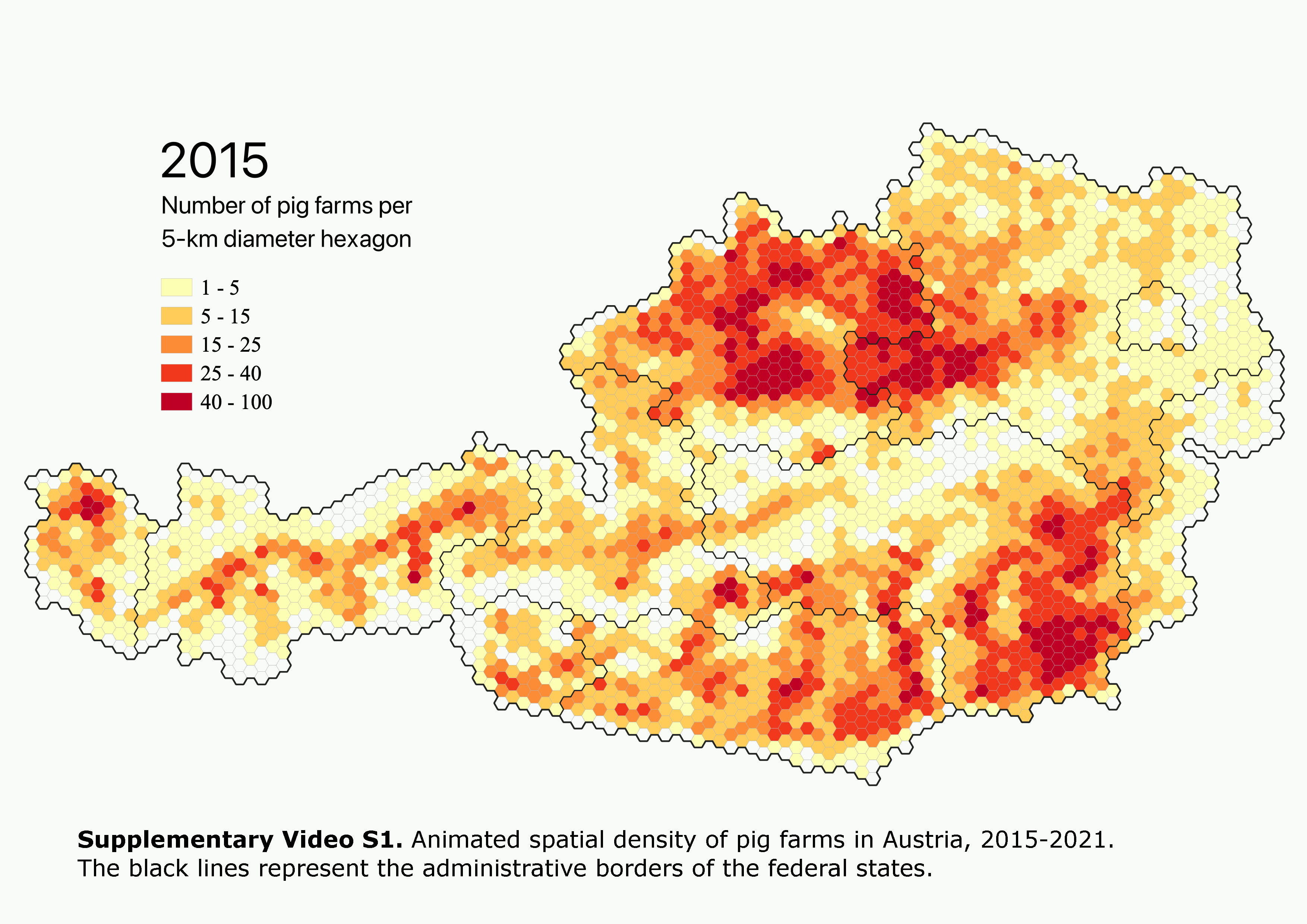

Supplement: Supplementary file 9 — Supplementary Information 9. [file 41598_2023_36596_MOESM9_ESM.gif]
